# Trust Matters: A Qualitative Analysis Of Medical Mistrust Among Older Black Adults

**DOI:** 10.1093/geroni/igaf122.4223

**Published:** 2025-12-31

**Authors:** Channing Tate, Denae Gerasta, Tsion Shiferaw

**Affiliations:** University of Colorado Anschutz, Aurora, Colorado, United States; University of Colorado Anschutz, Aurora, Colorado, United States; St Joseph’s Hospital Denver, Aurora, Colorado, United States

## Abstract

Medical mistrust is a major social driver of health and a persistent barrier to equitable healthcare for Black Americans. Historically, Black communities have been excluded from quality care through systemic racism and unethical practices like the Tuskegee Syphilis Study. These experiences have created deep-rooted mistrust in healthcare systems and providers. This study explores how older Black adults experience and conceptualize trust in healthcare. We focused on how both past and current experiences influence healthcare-seeking behaviors and identified preferred provider characteristics that promote trust. A purposive subsample of 12 Black adults aged 65 and older was drawn from a larger mixed-methods. Semi-structured interviews explored personal past and present experiences with the healthcare system, perceptions of trust and mistrust, and factors shaping care engagement. Thematic analysis identified multilevel determinants—individual, organizational, and systemic—that influence trust in both patient-clinician interactions and broader healthcare contexts. Participants described trust as multidimensional and evolving, shaped by past and ongoing exposure to systemic racism, discrimination, and structural barriers. Mistrust was reinforced by experiences such as microaggressions, dismissive treatment, and a perceived lack of respect. Conversely, trust was strengthened when providers showed competence, cultural humility, honesty, and respect for autonomy. Participants emphasized their own agency, often engaging in self-advocacy, information-seeking, and shared decision-making. These findings highlight the importance of addressing both structural inequities and individual needs. While systemic racism has fueled mistrust, consistent, respectful, and culturally responsive care can help rebuild trust and improve healthcare engagement among older Black adults.

